# The impact of drug allergy on planetary health and sustainability

**DOI:** 10.5415/apallergy.0000000000000229

**Published:** 2025-09-10

**Authors:** Bernard Yu-Hor Thong, Ruby Pawankar

**Affiliations:** 1 Department of Rheumatology, Allergy and Immunology, Tan Tock Seng Hospital, Singapore; 2 Department of Pediatrics, Nippon Medical School, Bunkyo-ku, Tokyo, Japan

Adverse drug reactions account for up to 3% to 6% of all hospital admissions and in 10% to 15% of hospitalized patients, resulting in morbidity, prolonged hospitalization, and risk of mortality [1]. Drug allergy is estimated to account for 1% to 2% of hospital admissions and 14% of emergency department visits, of which 0.6% are drug-induced anaphylaxis that lead to hospitalizations in 15% of cases [2].

There has been growing interest in the role of environment, sustainability, and governance (ESG) in the field of clinical immunology and allergy, in part due to the close interactions between the human genome, our environment, and the expression of allergic and immunologic disorders. One Health refers to the integrated, unifying approach that aims to sustainably balance and optimize the health of humans, animals, and the ecosystems, while recognizing that the health of humans, domestic and wild animals, plants, and the wider environment (including ecosystems) are closely linked and interdependent [3–5]. Planetary health refers to the achievement of the highest attainable standard of health, well-being, and equity worldwide through judicious attention to the human systems—political, economic, and social—that shape the future of humanity and the Earth’s natural systems that define the safe environmental limits within which humanity can flourish [6]. Greenhouse gas (GHG) emissions adversely impact planetary health and, consequently, human health. The health care sector contributes to more than 4% of net GHG emissions. The numerous factors in health care that contribute to these emissions include consumables, which require large amounts of raw materials and energy to produce; plastic disposal (contributing to plastic pollution), drug products and other chemicals; as well as global travel for meetings, incentives, conferences, and exhibitions (M.I.C.E). Increased use of telemedicine, virtual/hybrid conferences, and green chemistry helps raise awareness of and mitigate the negative impacts of the health care system (including health care research) on planetary and human health [7, 8].

Sustainability refers to the capacity to maintain or endure, focusing on the interplay of environmental, social, and economic factors. Protecting the biodiversity of plant and animal species is critical for sustainability for maintaining the natural balance of ecosystems. Setting sustainable development goals helps businesses work toward meeting the needs of the present without compromising our future generation. ESG is a tool that uses a standardized framework that enables investors and other stakeholders to assess the environmental and social impacts of a company, as well as its corporate governance practices. The “Environmental” component focuses on GHG emissions, energy efficiency, usage of renewable energy, waste management, and water conservation. “Social” sustainability involves managing and identifying business impacts on employees, workers, customers, and local communities. Social equity is the commitment to promote fairness and equality in its relationships, such as human rights, employee diversity, and labor standards, to improve relationships with people, communities, and society. The corporate “governance” component considers how a business is run, board diversity, inclusivity, compensation, risk management, and ethics.

Drug allergy may be related to ESG in several ways. Firstly, the misdiagnosis of drug allergy, where inaccurate drug allergy labels, especially to penicillins [9] deprive patients not just of the index penicillin, but potentially other non-penicillin betalactams, like cephalosporins. The misunderstanding of betalactam cross-reactivities [10, 11] often leads to the inevitable use of broad-spectrum antibiotics, increasing the risk of antimicrobial resistance like carbapenem-resistant organisms [12], adverse events like pseudomembranous colitis, and excessive use of fluoroquinolones and macrolides.

Secondly, drug allergy diagnostic testing is often not as simple as it may appear. Clinical features of the index reaction will direct the choice of in vivo and in vitro tests, each of which is associated with different sensitivities, specificities, positive and negative predictive values depending on the drug and the type of test [13]. Testing also requires infrastructure (eg, physical testing facility, equipment for resuscitation in the event of a systemic reaction during provocation testing), resources (eg, trained medical and nursing manpower), systems and processes, clinical governance, and enterprise risk-management (eg, for drug provocation tests and desensitization) [14]. In vivo tests like skin tests and patch tests require a robust supply chain for the procurement of raw material (eg, active drug ingredients and excipients), and in vitro tests require laboratory reagents and validated test kits. Over-labeling individuals with “multiple drug allergies” results in the potential need for multiple episodes of drug provocation tests [15], to improve their quality of life by removing inaccurate drug allergy labels [16]. The complex processes and resources spent delabelling inaccurate labels could potentially have been avoided in the first place through proper and careful evaluation before labelling a drug allergy.

As such, risk stratification of penicillin allergy labels into low- versus high-risk using the PEN-FAST tool [17] has facilitated penicillin allergy delabelling with direct oral challenge [18, 19] in both children and adults [20], in allergist and nonallergist-led clinics, extending to nurse- [21] and pharmacist-led [22] clinics in collaboration with primary care [23], using carefully structured protocols. A cephalosporin allergy clinical decision rule [24] has also recently been developed and validated where like PEN-FAST, a CEPH-FAST score of less than 3 is associated with a high negative predictive value and could be used by clinicians and antimicrobial stewardship programmes to identify patients with low-risk cephalosporin allergies at the point of care, following local validation, who could proceed to direct oral challenge or use non-cross-reactive cephalosporins.

Artificial intelligence (AI) tools [25, 26] may help clinicians to develop predictive models in drug allergy, given the heterogeneity and complexity of clinical phenotypes and mechanisms involved, the limitations of in vitro tests, and the associated risk of in vivo tests. Machine and deep learning, and artificial neural networks are emerging as powerful tools that could provide reliable and optimal predictive models and scores for clinical diagnosis, prediction, and precision medicine in different types of drug allergy, facilitating appropriate classification of patients [27]. Extensive data collection and curation are crucial for models’ development, which need effort and collaboration between multiple centers worldwide. On the contrary, few realize that AI and its applications in digital health, bioengineering, and society have significant material impacts on the environment owing to AI’s vast energy demands and energy consumption, carbon footprints, and water usage to cool data centers and generate electricity to power the data centers. The environmental footprints of AI remain under appreciated and inadequately acknowledged, particularly in this era of climate emergency and ongoing threats to planetary energy and water supplies, impacting planetary health. Nonetheless, the judicious use of AI in tandem with clinical judgement and knowledge accrued from clinical practice and experience, remain important in the recognition of severe cutaneous adverse reactions [28] and novel adverse effects of newer therapeutics for example biologics, small molecules, immune-checkpoint inhibitors [29]; and cell tissue and gene therapy products [30], applying recent knowledge of the immunopathogenesis of drug hypersensitivity [31] beyond traditional immunology teaching.

## Conflicts of interest

The authors declare no conflicts of interest.

## Author contributions

Bernard Yu-Hor Thong: Conceptualization and writing and review of manuscript. Ruby Pawankar: Review of manuscript.
